# Frontotemporal Dementia: Dilemma in Discrimination From Similarly Presenting Neurological and Psychiatric Conditions

**DOI:** 10.7759/cureus.28166

**Published:** 2022-08-19

**Authors:** Neil S Kidambi, Joyce Meza-Venegas, Luba Leontieva

**Affiliations:** 1 Psychiatry, State University of New York Upstate Medical University, Syracuse, USA

**Keywords:** neuroimaging and dementia, cognitive impairment and dementia, behavioral and psychological symptoms of dementia (bpsd), progressive dementia, early-onset dementia

## Abstract

Frontotemporal dementia (FTD) is the most common cause of neurocognitive decline, second to Alzheimer’s disease (AD) and Lewy body dementia. Its presence offers a unique challenge to physicians trying to detect cognitive deficits, as it not only arises in middle age but also can be misdiagnosed as a primary psychiatric disorder. The following case describes the clinical course of a 50-year-old male with a recent history of sporadic visual and auditory hallucinations, followed by a gradual decline in cognitive function including declining memory, apathy and behavioral disinhibition, and social functioning, which are suggestive of FTD-type. Apart from the gradual decline of his cognitive function, the patient had multiple clinical encounters, during which he was misdiagnosed with schizophrenia. Furthermore, the report showcases the handful of conditions that FTD can be misdiagnosed and discusses the thorough clinical/psychological examination and investigations to be done to arrive at FTD.

## Introduction

Frontotemporal dementia (FTD) manifests as a gradual devolution of higher functions, particularly language and or social behaviors, with onset as young as 30, although there is a considerable increase in the number of cases after the age of 50 [1]. It is a group of conditions encompassing a few distinct entities, namely, behavioral variant FTD (bvFTD) which involve deficits in emotional expression and social skills, semantic variant primary progressive aphasia (svPPA) is characterized by deficits in conceptual learning and identification, and nonfluent variant progressive primary aphasia which involves specific deficit targeting words and word output [1,2].

Of the three variants, bvFTD is the most common and by far the most serious [3]. This variant is characterized by a gradual regression and disinhibition of complex social and personal behavior including lewd acts (i.e., public urination), impulsive actions (i.e., reckless spending), and reprehensible language. Their emotional responsiveness to others undergoes a decline, and they may display apathy toward their loved ones. With progression, they will demonstrate perseveration: following the same routine or using the same words when questioned [2].

Other conditions with similar features that must be considered include:

Late-onset psychosis (e.g., late-onset schizophrenia) is important to distinguish from bvFTD. While late presentations normally occur after the age of 60, some variants can present between 40 and 60 years. Amid psychosis, individuals will lack insight and exhibit disorganized behavior [4]. They exhibit mood lability as well as auditory and visual hallucinations, with a family history of psychoses, which increases predisposition. While a thorough clinical examination can delineate both conditions, a dilemma in discrimination can arise when the patient presents with a genetic variant, C9oRF72, in which there are neuropsychiatric symptoms such as mania and irritability, but there is no family history [3-5].

The frontal (behavioral) variant of AD (fvAD or bvAD) often overlaps with the bvFTD with regard to the clinical presentation. Patients will experience a slow decline in executive function with disinhibition of social behavior, blunting of affect, and memory deficit [6]. Given the similarities in clinical presentation, it is imperative to distinguish this from the frontal variant with a series of tests including episodic memory tests as part of neuropsychological testing and imaging modalities (i.e., MRI or FDG-PET) [6-7].

Chronic traumatic encephalopathy (CTE) is also a diagnosis of exclusion, given that it is diagnosed post-mortem via histopathology of the brain. However, the clinical presentation raises interesting questions because of its close resemblance to the course of other major causes of dementia as well as a primary psychiatric disorder. In addition to neurological symptoms such as dizziness and headaches and parkinsonian-like features, the patient experiences a range of other features like emotional instability leading to unprovoked aggression, paranoid delusions, frequent disorientation, and confusion [8,9].

## Case presentation

The patient is a 50-year-old low-functioning male admitted with confusion and disorganized speech. Prior to his admission, the patient was of a lower socioeconomic background, having worked as a garbage collector for 20 years. Additionally, during this time, the patient worked as a boxing trainer for about 10-15 years. His presentation proved challenging given his past psychiatric diagnosis of schizophrenia and prior clinical documentation that pointed to a psychotic presentation. However, psychosis was ruled out following a thorough psychiatric, neurologic, and neuropsychological evaluation, and the major neurocognitive decline was concluded as the cause of the patient’s presentation, with a possible diagnosis of FTD.

The patient’s first psychiatric visit was at age 45, for both depressive- and anxiety-related symptoms. His wife had left him three months prior, and he had made threats of self-harm. He had episodes during which he would isolate himself and often retreat into his room with little self-maintenance to the point of urinating on himself. At the time, the patient was diagnosed with major depressive disorder and prescribed duloxetine. Following this visit, the patient was following up with outpatient therapist, and by all accounts appeared to be adjusting to his living situation.

About four months later, the patient had been arrested after his wife reported that he held her at gunpoint for several hours. He was apathetic, disorganized, and behaviorally disinhibited, having been completely naked while the event transpired, and discharged his gun in her direction. At several points during this event, the patient urinated on his wife.

During the next three years, the patient had multiple hospital visits for a worsening of his depressive symptoms but failed to comply with medication and failed to follow up with a physician. At age 48, the patient began demonstrating hallucinations: 1) he called his daughter from an undisclosed location to report that his grandchildren were sitting in his car when they were actually not. 2) He was found at a gas station by police, hallucinating bears attacking children. He was subsequently brought to a hospital where his urine drug screen and ethanol level were negative. Additionally, the patient’s supervisor at his workplace noticed that he was disoriented and failed to recognize where he was. He was required to get a mental health evaluation before he could work again. However, the patient did not appear for the evaluation and voluntarily left his job. The patient had also been in multiple failed relationships and had begun living out of his car.

About two months later, the patient moved in with his daughter for a short period of time before being asked to leave as he was irritable, and appeared disruptive. Following this, the patient alternated between staying with a friend and homeless shelters. However, he experienced a furthering of his behavioral disinhibition, where he was often fighting with others on the street and would wander into traffic, possessing no memory of the incident afterward. He had multiple admissions to the ED where he was diagnosed with acute psychosis which raised a concern for possible schizophrenia. He was discharged with escitalopram, gabapentin, and aripiprazole. In the meantime, he was followed by neurology, where he was found to have a Montreal Cognitive Assessment (MOCA) of 13/30 and was diagnosed with an unspecified neurocognitive disorder. His CT scan and EEG at this time showed no abnormalities.

At the age of 50, he was readmitted to the hospital for unspecified psychosis and unspecified dementia when he was found wandering after being missing for 5 days. Brain CT and brain MRI were normal, urine drug screening (UDS) was negative, and B12 was within normal limits. At the time, a preliminary diagnosis of dementia was established and he was discharged on memantine and donepezil. After the discharge, he did not follow up and started living in a men’s shelter where he was expelled shortly after attacking another resident.

 Social history

The patient’s early childhood was marked by parental separation, neglect, and reports of physical abuse. His parents got divorced when he was 6 and he was then cared for by his alcoholic mother. According to his ex-wife, the patient’s mother had several relationships, during which the patient was exposed to alcohol as well as experienced physical abuse. He met his ex-wife as a teenager and soon had a daughter and a son. The patient’s baseline was “moody” and he was never really close to his children. There was a traumatic episode when the patient was in his 20s when someone broke into their house and assaulted both his wife and infant son. Following this incident, the patient’s behavior changed dramatically. He began to be verbally and physically abusive to his wife and his children, would break things in angry bursts, and spend money excessively. When he was out with friends, he would drink excessively and would get into fights. He did not use illegal drugs.

Current hospital course

**Figure 1:**
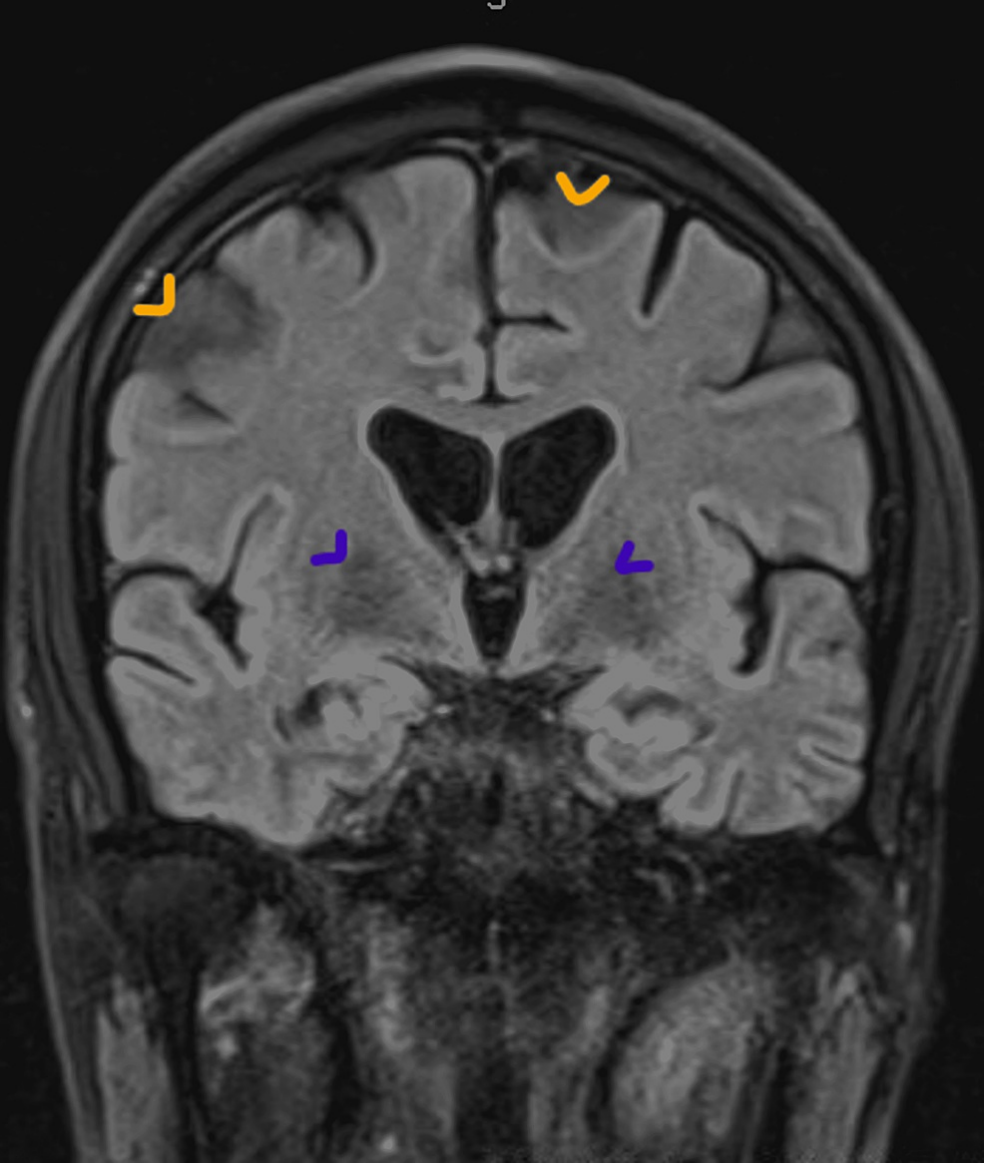
Patient's MRI. T2/FLAIR shows nonspecific hyperintensities in the periventricular (purple arrows) and subcortical (yellow arrows) regions, which is otherwise unremarkable. FLAIR: fluid-attenuated inversion recovery

Presently, he is not oriented to place, time, or the purpose of the unit. Spontaneous speech is characterized by a slow rate owing to word-finding pauses, but there is no frank agrammatism. His speech is incoherent and disorganized most of the time. The effect is apathetic and he appears to be blunted. His thought process is impoverished, incoherent, disorganized, and tangential. Interestingly, with the assistance of others, he can recall concrete details but his poor recall ability prevents him from remembering surrounding details. The patient stands and sits normally without effort and his gait is normal; rigidity, tremor, or fasciculations are not seen. His balance is normal, both when he stands and walks; the pull test elicits normal righting reflexes. There is no Romberg sign. CT, CSF Lyme antibodies, and antinuclear antibodies (ANA) are all unremarkable. CSF pathogen panel and CSF and serum dementia autoimmune panels are negative. Routine EEG is within the normal range. Lumbar puncture is performed and has an elevated CSF protein at 88, with cell count and glucose levels within normal levels. CSF analysis of beta-amyloid and phosphorylated tau and total tau are consistent with Borderline Alzheimer’s dementia (Table 1). His MRI without contrast delineated hyperintensities in the periventricular and subcortical regions, which are otherwise nonspecific findings (Figure 1). His MOCA a month from admission reveals a score of 9/30, consistent with global deficits in his cognition.

**Table 1:** Summary of CSF panel pertinent to the workup of Alzheimer dementia. Abnormal values are bolded. Not consistent with AD; P-Tau < 54 pg/mL and ATI > 1.2, borderline; P-Tau 54-68 pg/mL and/or ATI 0.8-1.2, AD; P-Tau > 68 pg/mL and ATI < 0.8 ATI: A beta 42/Tau protein index

Test Variables	Technical Result
A beta 42	329.6 pg/ml
T-Tau	579.2 pg/ml
P-Tau	74.8 pg/ml
ATI	0.36

A neuropsychological assessment revealed a major neurocognitive disorder (dementia) of unclear etiology. On a measure that estimates premorbid intellectual ability, the patient performed in the borderline range (Test of Premorbid Functioning (TOPF) = 73). With respect to global cognitive functioning: he was not oriented to time, and he was partially oriented to place. Concerning attention/concentration, basic auditory attention was in the borderline range with four digits correctly sequenced in the forward order on the Wechsler Adult Intelligence Scale 4th Ed (WAIS-IV). On executive functioning, phonemic fluency and verbal abstract reasoning were borderline while nonverbal abstract reasoning was extremely low. With regards to language, confrontation naming was borderline, with word-finding difficulty and circumlocution on this task. Additionally, there were occasional paraphasic errors and semantic fluency was extremely low secondary to loss of a set and tangential responses. On visuospatial assessment, the construction of simple to complex geometric figures was extremely low. Lastly, with regards to memory, recall, and recognition discrimination was low due to the inability to complete the recognition discrimination portion of the test because of concrete, tangential thinking.

Based on his clinical presentation, neuropsychological evaluation, and CSF analysis of beta-amyloid and phosphorylated tau and total tau, the patient’s diagnosis is most consistent with a major neurocognitive disorder, likely FTD vs a variant of AD.

## Discussion

This patient’s presentation proved challenging given his past psychiatric diagnosis of schizophrenia as well as abundant clinical documentation that alluded to a psychotic presentation. However, following a thorough psychiatric, neurologic, and neuropsychological evaluation, psychosis was ruled out, while the major neurocognitive decline was deemed the cause of the patient’s presentation, with a possible diagnosis of FTD.

The complexity of this case lies in the overlap of symptoms between bvFTD and psychiatric disorders. Both conditions have similarities in their presentation of features like mania, such as irritability, and agitation, as well as psychotic symptoms like delusions and hallucinations. However, through psychiatric examination, the presence of emotional distress is observed in psychiatric disorders, as opposed to bvFTD, where the patients are apathetic. Next, nuanced changes such as the fluctuation of the symptoms and a lack of insight (understanding of debility of their condition) are considerably more frequent in bvFTD compared to those with psychiatric disorders [3-5]. Additionally, in the case of schizophrenia, late-onset variants can present with a functional decline and/or symptoms such as apathy, catatonia, or disorganization before the age of 60. MRI may define changes in the contour of the brain, specifically, atrophy of the frontal and temporal regions. This is a limiting factor for the diagnosis of bvFTD because atrophic changes to these regions do not necessarily have to be present, FDG-PET, a more specific imaging modality, can detect decreased O_2_ metabolism by the prefrontal and anterior temporal lobe, while the occipital lobe retains its metabolic capability [5,6].

Consideration should be given to the frontal variant of AD (fvAD), as there can be considerable overlap in the clinical presentation between fvAD and bvFTD. The key to differentiation lies in clinical presentation, laboratory findings, and neuro-imaging. First, in AD, an elevated A-beta-42, T-Tau, and P-Tau proteins would be elevated in the CSF, which was also found to be elevated in the patient. In fvAD, clinically, however, there is a milder behavioral profile compared with bvFTD, with less compulsivity and hyperorality but a greater prevalence of neuropsychiatric symptoms, such as agitation, delusions, and hallucinations. Furthermore, bvAD presents with greater executive dysfunction compared with bvFTD [7]. In neuroimaging, the relative pattern of involvement of anterior brain structures helps in differentiating patients with bvFTD from those with typical Alzheimer’s dementia which usually targets posterior regions of the brain, including the posterior temporal and parietal lobes [6]. Regarding the patient, he exhibited agitation, communication, and memory deficits, but unlike fvAD, there was relative preservation of social cognition. Additionally, he experienced a few episodes of visual hallucinations but did not appear to have any delusions.

As this patient has a known boxing history, concerns for CTE must be addressed. The clinical features of CTE resemble the ones present in bvFTD which are deterioration in personality, social comportment, and cognitive functioning [8]. BvFTD usually presents insidiously with changes in personality, interpersonal conduct, and emotional regulation. Apathy, disinhibition, repetitive stereotypical behaviors, or perseveration are frequently observed as well. Patients affected with CTE usually have well-preserved language and visuospatial abilities but higher-order, executive functions tend to be adversely affected [8,9]. This is similar to the clinical presentation in traumatic brain injury (TBI) where apathy, loss of social norms, and decreased empathy seen in bvFTD are frequently seen due to frontal lobe degeneration [9]. Regarding the patient, there are limitations regarding the history; while the duration of boxing is known, his level of exposure to head trauma is not known: how much he boxed himself versus how much he trained others. Additionally, while the patient does have relative preservation of speech, his communication skills are severely limited. He also does not exhibit repetitive behaviors or perseveration.

## Conclusions

Neurocognitive decline poses an increasing challenge for global public health. While there may be a myriad of presentations, there must also be a conscious effort to recognize the neurocognitive decline in young patients, the different causes, especially FTD, and the similarities between these different causes. Despite the best efforts of individuals to create effective clinical tools for the diagnosis of FTD, there is still disagreement over the sensitivity of said tools. More effort should be put forth to streamline criteria for the evaluation of FTD.
